# Pausing for thought: Disrupting the early transcription elongation checkpoint leads to developmental defects and tumourigenesis

**DOI:** 10.1002/bies.201200179

**Published:** 2013-04-10

**Authors:** Barbara H Jennings

**Affiliations:** Transcriptional Regulation Group, UCL Cancer InstituteLondon, UK

**Keywords:** DSIF, NELF, promoter proximal pausing, P-TEFb, Spt5, transcription elongation

## Abstract

Factors affecting transcriptional elongation have been characterized extensively in in vitro, single cell (yeast) and cell culture systems; however, data from the context of multicellular organisms has been relatively scarce. While studies in homogeneous cell populations have been highly informative about the underlying molecular mechanisms and prevalence of polymerase pausing, they do not reveal the biological impact of perturbing this regulation in an animal. The core components regulating pausing are expressed in all animal cells and are recruited to the majority of genes, however, disrupting their function often results in discrete phenotypic effects. Mutations in genes encoding key regulators of transcriptional pausing have been recovered from several genetic screens for specific phenotypes or interactions with specific factors in mice, zebrafish and flies. Analysis of these mutations has revealed that control of transcriptional pausing is critical for a diverse range of biological pathways essential for animal development and survival.

## Introduction

Cells within a mature animal differ dramatically in their size, shape, function, longevity and ability to keep dividing even though, with few exceptions, every cell contains the same set of genes. The great diversity of cells found in animals is a consequence of different cell types expressing different profiles of genes during cell fate determination and differentiation. Failure of gene regulation usually has catastrophic effects on the developing embryo, and in adult life leads to disease, including cancer.

All gene expression is controlled at the level of RNA polymerase recruitment and successful formation of the pre-initiation complex (PIC). Obviously, if RNA polymerase cannot bind to the transcription start site of a gene, then no RNA can be transcribed. However, transcription in eukaryotes may be regulated at several additional levels, including elongation, processing, termination and export from the nucleus.

It had been established for over 30 years that transcription elongation may be a rate-limiting step in gene expression, but it is only in the past five years or so that the prevalence and importance of elongation control has been recognized (recent reviews include 1–4). Historically, the best-studied example was regulation of the genes encoding the heat shock proteins (Hsp) in *Drosophila*. In the absence of heat shock, transcriptionally engaged RNA polymerase II (RNAP II) accumulates just downstream of the transcriptional start sites (TSSs) of *Hsp* genes and is associated with short 20–60 nucleotide long nascent RNAs 5, 6. This phenomenon is often described as promoter proximal pausing. Upon heat shock, activating factors trigger the release of RNAP II from promoter proximal pausing, and there is rapid increase of full-length transcripts produced from *Hsp* genes 5, 6.

Another well-studied example of elongation control is transcription of the HIV provirus 7–9. Transcription of HIV is a critical step in the virus's life cycle. HIV provirus is integrated into the host chromatin where it becomes subject to transcription by host RNAP II to replicate the virus. In the absence of the Tat activator protein (encoded by HIV), RNAP II can initiate transcription efficiently and clears the promoter, but synthesizes short non-polyadenylated transcripts 10. When Tat is present, it recruits factors that activate transcription elongation, including Positive Transcription Elongation Factor b (P-TEFb; see below) and full-length transcripts of the virus are made. Inhibition of transcription elongation has been one strategy investigated as a therapy for HIV infection.

Other examples of genes showing promoter proximal pausing emerged into the literature sporadically, including *c-myc* and *c-fos* 11–13. Then, starting in 2007, the arrival of new technologies permitting whole genome analysis led to a slew of studies of RNAP II recruitment and transcript production. These studies revealed that promoter proximal pausing is a feature of many metazoan genes 14–16.

RNAP II typically displays an approximately uniform distribution of binding across transcription units in yeast, consistent with a model in which RNAP II experiences no regulatory barriers after transcription initiation 17. However, in higher eukaryotes RNAP II binding is concentrated near the transcription start site of many genes consistent with promoter proximal pausing. Guenther et al. 15 demonstrated that while approximately 75% of protein coding genes in human embryonic stem cells experience transcription initiation, only about half of these genes produce detectable full-length transcripts. Furthermore, two genome-wide screens for promoter proximal paused RNAP II in *Drosophila* revealed that approximately 20% of genes in S2 culture cells, and 10% in early embryos, had initiated transcription but were transcriptionally paused 14, 16. More recent studies have confirmed that the majority of RNAP II associated with the promoters of *Drosophila* genes is paused and this is a checkpoint that is widely used to regulate transcription 18, 19.

**Figure 1:**
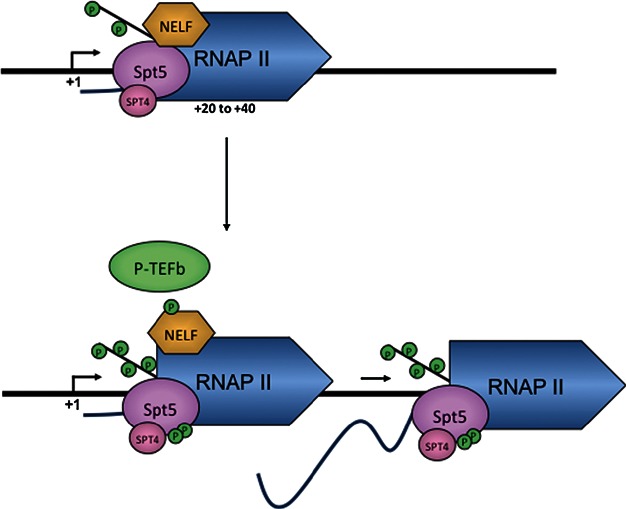
Model of promoter proximal pausing and release. Spt4 and Spt5 (DSIF) and NELF act together to inhibit transcript elongation beyond ∼20–60 nucleotides. For further elongation to occur, P-TEFb must phosphorylate specific residues in NELF, Spt5, and the long C-terminal domain (CTD) of RNAP II. This induces the dissociation of NELF, the switch in Spt5 from being a repressor to an activator of transcription, and production of the full-length transcript by RNAP II.

The current model for RNAP II promoter proximal pausing and release is largely based on in vitro studies using human cell lysates (Fig. 1). Briefly, two protein complexes, one containing Spt4 and Spt5 (often referred to as ‘DSIF’) 20 and the other known as Negative Elongation Factor (NELF) 21, act together to inhibit transcript elongation beyond ∼30 nucleotides 2, 22, 23. For further elongation to occur, the Cdk 9 kinase subunit of P-TEFb must phosphorylate specific residues in NELF, Spt5 and RNAP II. This induces the dissociation of NELF from the polymerase complex, the switch in Spt5 from being a negative to positive regulator of transcription and production of the full-length transcript by RNAP II. Spt5 tracks along with the RNAP II elongation complex until transcription termination. Recent structural studies have shown that the ‘NGN’ domain of Spt5 sits over the DNA and RNA bound in the active site of RNA polymerases, where it can directly control the rate of transcript elongation 24, 25.

Many reviews have recently been written about the molecular mechanisms controlling promoter proximal pausing 1–4, 22, 23, 26–28, but the biological consequences of aberrant elongation control have been largely overlooked. Not surprisingly, null mutations in core factors regulating the transition into elongation are often lethal to the cells that carry them, but some are not, and other less severe aberrations also provide insight into the role of promoter proximal pausing in animals. The remainder of this essay will highlight results from animal studies of mutations in core factors controlling elongation and consider what they reveal about the role of these factors in biology.

## Spt5 function during animal development

Spt5 is involved with all transcription; it is conserved across all three domains of life (eukaryotes, archaea and bacteria) and interacts with RNA polymerases I, II and III 29. Given that Spt5 has such a ubiquitous role in transcription, it is perhaps surprising then that several mutations in Spt5 have been recovered from genetic screens for very specific developmental defects or phenotypic interactions. Thus, Spt5 may provide a junction between contextual transcriptional regulators and RNAP II.

The first mutant allele of *Spt5* (*foggy*^*m806*^) was recovered from a genetic screen for mutations affecting neuronal development in zebrafish 30. Homozygous *foggy*^*m806*^ fish look relatively normal but have neuronal defects that ultimately prove lethal, including a deficit of dopamine-containing neurons and corresponding excess of serotonin-containing neurons 31. *foggy*^*m806*^ is a missense mutation that leads to an amino acid substitution (V1012D) in the very C-terminal region of Spt5, which is conserved only amongst higher eukaryotes. Subsequently, null alleles of *Spt5* were characterized in zebrafish that had originally been generated in a large-scale screen for mutations affecting embryogenesis 32, 33. Fish homozygous for *Spt5* null alleles have additional phenotypes with respect to *foggy*^*m806*^ homozygotes, including reduced pigmentation, short tails, small ears and pericardial oedema 33.

There is a maternal component to Spt5 expression in zebrafish, thus homozygous null animals do contain some residual wild-type protein. However, the phenotypes observed in these embryos as the maternal component diminishes are highly specific and reproducible, indicating that the expression of a specific subset of genes during development is more sensitive to a reduction in Spt5 availability than others. Subsequently, expression profiling of over 10,000 protein coding genes in zebrafish embryos 24 hours postfertilization identified that only 5% of genes were differentially expressed between null mutants and their wild-type siblings 34. Thus in zebrafish, a small subset of genes is highly sensitive to Spt5 levels during embryogenesis, suggesting that they may represent direct targets of regulation by Spt5. A similar study using morpholino oligonucleotide-mediated knockdown of *Spt5* coupled to time-course microarray analysis of early zebrafish embryos indicated that Spt5 plays an erythroid-specific role in early embryogenesis through the induction of *gata1* gene expression 35.

A further zebrafish allele of *Spt5* (*fh20*) was isolated from a genetic screen to identify genes that control the posterior migration of facial branchiomotor neurons in the zebrafish hindbrain 36. The sequence change in the *fh20* allele causes a splicing defect, leading to a mix of correctly and incorrectly spliced mRNA and a hypomorphic phenotype. *Spt5* null facial branchiomotor neurons survive to at least five days postfertilization while failing to migrate posteriorly in a wild-type host 36. Thus, Spt5 appears to play an important role in branchiomotor neuron migration in zebrafish.

The N-terminal region of Spt5 (NSpt5: lacking the repeats phosphorylated by P-TEFb) acts in a dominant manner to disrupt development when expressed in zebrafish embryos 37. This variant impairs the repressive function of Spt5 in vitro and leads to de-repression of *hsp70* in the absence of heat shock in vivo 37.

The *Drosophila Spt5*^*W049*^ allele was recovered from a genetic screen for maternal factors that affect anterior-posterior patterning during embryogenesis 38. When homozygous in the maternal germ line (such that all Spt5 deposited in the embryo by the mother is the mutant variant), *Spt5*^*W049*^ leads to defects in segmental patterning of the embryo. The effects of *Spt5*^*W049*^ are gene specific, since expression of the gap genes is normal while expression of two of the three primary pair-rule genes, *even-skipped* (*eve*) and *runt* (*run*), is aberrant. Furthermore, enhancer-reporter constructs reproducing specific stripes of *eve* expression are affected differentially by *Spt5*^*W049*^: expression driven by the stripe 2 enhancer is weak but broadened, while expression of stripe 3 appears wild-type. These results indicate that Spt5 is sensitive to the different combinations of *trans*-acting factors that drive expression of stripe 2 and stripe 3.

Remarkably, the *Spt5*^*W049*^ missense mutation maps to the same region of Spt5 as the *foggy*^*m806*^ mutation in zebrafish, implicating this domain of Spt5 in interactions with contextual factors that regulate its activity during development. Assays performed in nuclear extracts demonstrated that both the Foggy^m806^ and W049 protein variants have a diminished ability to inhibit transcription prior to the phosphorylation events of the P-TEFb checkpoint 31, 38. We observed a loss of repression of *eve* expression in the early embryo; stripes of eve pair-rule protein are broadened or fused in late, cellularizing *Spt5*^*W049*^ embryos when the pattern should be fully resolved into seven distinct stripes. Thus, the inhibitory activity of Spt5 on transcription prior to the P-TEFb checkpoint has a role in repression of *eve* in the early embryo. In wild-type embryos, a subset of the cells that repress *eve* expression during the seven stripe stage re-activate *eve* expression around 30 minutes later during gastrulation to form interstripes. Thus, the repressive mechanism involving Spt5 is rapidly reversible.

Two null alleles of *Drosophila Spt5* were recovered in a genetic screen for factors that modify *Presenilin*-dependent *Notch* phenotypes 39. These mutations (when heterozygous) enhance loss-of-function *Notch* phenotypes, indicating that Spt5 is required to activate gene expression in response to Notch signalling. Animals homozygous for the *Spt5*^*W049*^ missense mutation 38 and null alleles (B.H.J. unpublished data) show diminished activation of heat shock gene expression in vivo. Spt5 mediates repression of the *eve* locus and activation of heat shock gene expression, thus Spt5 clearly has both positive and negative effects on transcription in vivo dependent on context.

Very recently, further alleles of *Drosophila Spt5* have been recovered from a genetic screen for factors mediating dosage compensation 40. In *Drosophila*, males (XY) make additional transcripts from their single X chromosome, to match the amount transcribed from females (XX). The increased transcription in males is dependent on the MSL complex, which contains at least five different proteins and two non-coding RNAs. Rather than localizing to the promoter or TSS, the MSL complex is found across the gene bodies of active genes 41 consistent with a model in which it acts during transcription elongation. *Spt5* genetically interacts with genes encoding components of the MSL complex and Spt5 protein physically interacts with MSL1. Thus, it seems likely that in the case of dosage compensation, Spt5's role is to promote active elongation across the gene body rather than in establishing the P-TEFb checkpoint 40.

## The NELF complex regulates pausing in higher eukaryotes

The NELF complex, which is made up of four subunits (NELF-A, NELF-B, NELF-C/D and NELF-E), is found only in higher metazoans; it is not found in yeasts or the nematode *Caenorhabditis elegans*. NELF is recruited to a large subset of genes 42 where it physically interacts with DSIF and RNAP II to establish promoter proximal pausing. Like DSIF, NELF has been implicated in both activation and repression of transcription; depletion of NELF in *Drosophila* S2 culture cells leads to both up and down regulation of target genes 42. This observation contributed to the model where NELF recruitment influences nucleosome positioning around the TSS and renders genes in a state where RNAP II can be rapidly recruited 42, 43.

Mutations in the human gene encoding the NELF-A subunit have been implicated in the pathogenesis of Wolf–Hirschhorn syndrome (WHS). WHS is caused by deletions of the distal part of the short arm of chromosome 4 that remove the *WHSC1* and *WHSC2* genes 44. *WHSC2* encodes NELF-A. The clinical presentation of WHS is complex and its severity correlates with the size of the chromosomal deletion. Clinical features include a distinctive craniofacial phenotype, growth and mental retardation, seizures and cardiac abnormalities. The precise contribution of *WHSC2/NELF-A* gene deletions to this syndrome are not yet understood due to the variability of the associated chromosomal aberrations that remove numerous genes, and the lack of good animal models 45.

NELF-B was recovered in a yeast 2-hybrid screen for factors that interact with breast cancer susceptibility gene BRCA1 and is sometimes referred to as COBRA1 (co-factor for BRCA1) 46. NELF-B inhibits the growth of estrogen receptor α (ERα) positive breast cancer cells in vitro 47 and NELF-B expression is reduced in several established breast cancer cell lines 48. A study of a cohort of breast ductal carcinoma samples from patients with known clinical outcomes revealed that a low level of *NELF-B* mRNA is associated with metastatic breast cancer 49.

Mice homozygous for a mutation in *NELF-B* have an inner cell mass deficiency and die at the time of implantation 50. Thus, NELF-B plays a critical role in early mouse embryogenesis.

NELF also plays a critical role during early *Drosophila* development 51. More than half of embryos derived from maternal germ line clones homozygous for a null allele of *NELF-A* cease to develop before the cellular blastoderm stage and display abnormal nuclear morphology. The remaining embryos gastrulate normally, however, they generally stop developing during germband retraction and exhibit head defects and incomplete dorsal closure. Embryos derived from maternal germ line clones of a hypomorphic allele of *NELF-E* show similar, but less severe developmental defects 51. Despite the obvious developmental defects, patterning and expression of endogenous segmentation genes, including *eve*, *fushi tarazu* (*ftz*), and *sloppy paired 1* (*slp1*) at the cellular blastoderm stage is apparently normal in both the *NELF* maternal germ line clone embryos, although the expression of some reporter transgenes derived from these genes is greatly diminished 51.

There is a reduction in activation of immune response pathways in larvae depleted of NELF by RNAi, supporting a role for NELF in gene activation 52. Many genes in the immune response pathways in *Drosophila* contain paused polymerases and are expressed at basal levels in the absence of pathogens 52. However, it is interesting to note that many of the genes encoding effector proteins at the end of the pathways [e.g. genes encoding antimicrobial peptides (AMPs) including *CecA1*, *DptB* and *AttA*] do not contain paused polymerases but do become rapidly and highly activated. Stimulus-sensing genes, which must always be expressed at some low level, are more likely to contain paused polymerases than effector proteins, which need only be expressed in response to an immune challenge 52. Thus, in this example, promoter proximal pausing represents a mechanism to precisely control levels of gene expression, rather than to switch gene expression on or off.

NELF localizes to the heat shock genes when they are not induced, where it collaborates with DSIF to establish pausing. Depletion of NELF-E in *Drosophila* salivary glands reduces the level of paused polymerase found on *Hsp70* 53. For many years, the presence of paused polymerases was proposed to contribute to the rapid rate of transcriptional induction upon heat shock so the observation that depletion of NELF does not affect the rate of heat shock gene induction perhaps came as a surprise 54. Loss of NELF delays the time taken for *Hsp* transcription to decrease down to basal levels after the heat shock stimulus has ceased. The presence of NELF somehow facilitates the dissociation of heat shock factor from target genes 54.

The immune and heat shock responses demonstrate that promoter proximal pausing is not an absolute requirement for rapid activation of transcription. Moreover, promoter proximal pausing often appears to suppress expression of inducible genes to basal levels when there is no stimulus present.

Although it is well established that DSIF and NELF act together, the biological consequences of perturbing DSIF and NELF activity are distinct from each other in *Drosophila*. It is not possible to make embryos from germ line clones of null alleles of *Spt5* or *Spt4* as complete loss of either of these factors is lethal to cells in *Drosophila* (B.H.J. unpublished result), whereas the NELF-A null clones do survive for a time 51. Disrupting the NELF complex does not alter patterning of the endogenous *eve* gene whereas *Spt5*^*W049*^ embryos lose repression of *eve* in interstripe regions 38. Furthermore, compromising Spt5 activity leads to a diminished induction of heat shock genes while compromising NELF does not affect induction, but does affect the rate of recovery back to basal expression levels.

## Direct regulators of P-TEFb activity control animal development and cancer pathogenesis

P-TEFb plays a critical role in the activation of all transcription and the regulation of its activity can be a rate-limiting step in metazoan gene expression 4, 26–28, 55, 56. It is made up of two subunits: Cyclin T and Cdk9. Cdk9 is a protein kinase whose targets include Spt5, NELF and RNAP II. A number of factors that interact directly with P-TEFb contribute to regulation of promoter proximal pausing. P-TEFb activity is regulated directly by its sequestration and release by an inhibitory complex (7SK snRNA/LARP7/HEXIM) and activation by association with Bromodomain-containing protein 4 (Brd4) or c-Myc, or by inclusion in ‘super elongation complexes’ (SECs).

### 7SK snRNP

The 7SK small non-coding RNA (snRNA) inhibits RNAP II transcription by binding P-TEFb and recruiting an RNA binding protein HEXIM (HEXIM1 or HEXIM2 in mammals) to block Cdk9 activity 27. The La-related protein (LARP7) and 7SK methyl phosphate capping enzyme (MePCE) are constitutive components of the 7SK snRNP. Together, they stabilize the RNA and may be involved in regulation of the release of P-TEFb. The association of P-TEFb with the 7SK snRNP complex is reversible and regulation of this association is a key mechanism to control P-TEFb activity in cells.

Mice homozygous for a targeted knockout of the *HEXIM1* gene (referred to as *CLP-1* in some publications) generally die before birth and have heart defects 57. Ectopic HEXIM1 expression in the mouse mammary gland decreased estrogen-driven ductal morphogenesis and inhibited the expression of Cyclin D1 58. Ligand-bound ERα regulates formation of the HEXIM1/P-TEFbcomplex in breast cells 58. The different HEXIMs in mammals may interact with different regulatory factors to generate context-specific regulation of P-TEFb activity 58.

*Drosophila* contains just one member of the HEXIM family, which, along with the 7SK snRNA, is ubiquitously expressed during development 59. Tissue specific knockdown of HEXIM in flies (using the *GAL4>UAS-RNAi* system 60) indicates that it is required for cell viability. Although LARP7 is expressed throughout *Drosophila* development, there are noticeable differences in levels in different cell types. For example, LARP7 is expressed strongly in the ommatidial clusters in the eye imaginal disc and a cluster of cells in the presumptive notum of the wing disc 59. Knockdown of LARP7 in zebrafish by morpholinos leads to severe developmental defects including altered axis formation and possible neurodegeneration 61.

### Brd4

Proteomic analysis of human proteins associated with mouse Brd4 revealed that Brd4 interacts with P-TEFb 62. Brd4 has been implicated in cell cycle control and DNA replication 63. Brd4 has a positive role in transcription by recruiting active P-TEFb complexes to acetylated chromatin in promoter regions 62.

The *Drosophila* orthologue of *Brd4* is encoded by *female sterile (1) homeotic* [*fs(1)h*; sometimes written as *fsh*]. Fsh is a Trithorax group protein, whose main action is to maintain gene expression. Binding of Fsh to promoter regions is predictive of transcriptional activity in *Drosophila* S2 cells 64. Loss of *fs*(*1*)*h* function results in segmental abnormalities including homeotic transformations in the progeny of mutant mothers 65.

### c-Myc

The c-Myc protein contains a transactivation domain that interacts directly with the Cyclin T subunit of P-TEFb to stimulate transcription by releasing paused polymerases 66–68. c-Myc regulates expression of many key genes controlling growth and proliferation during normal animal development, and plays a major role in cancer pathogenesis 69. The *c-myc* gene is one of the most highly amplified oncogenes isolated from human cancer 70. It has been shown in mouse ES cells that c-Myc stimulates P-TEFb activity to overcome pausing at many actively expressed genes, including genes driving cell proliferation 68. Thus, as a direct target of c-Myc function, the pausing checkpoint has a critical role in driving cell proliferation during both animal development and cancer pathogenesis.

### Super elongation complexes (SECs)

A small fraction of P-TEFb is present in SECs where the Cdk9 kinase is highly active 4, 26, 28. Mutations in a number of components of SECs have notable phenotypes in animals. In addition to P-TEFb, a SEC may include AFF1/AF4, AFF4, ELL1, ELL2, ENL and AF9. The composition of SECs varies and additional factors are probably involved, creating potential for regulatory diversity.

Key components of SECs have been identified as frequent translocation partners of the mixed lineage leukaemia (MLL) protein in leukaemia 26. MLL is a DNA binding transcription factor that is involved with maintaining active transcription and plays many important roles in development, which include haematopoietic stem cell development and maintenance. Chromosomal translocations that lead to in frame fusions of the *MLL* gene to other proteins are often recovered from acute and aggressive myeloid and lymphoblastic leukaemias. Five integral components of SECs (AFF1/AF4, AFF4, AF9, ENL and ELL1) are frequent translocation partners to MLL 71. Thus the pervading model is the N-terminal DNA binding domain of MLL fused to a SEC component will recruit the rest of the SEC and P-TEFb to MLL target genes to stimulate their expression above normal levels, which can cause leukaemia 26, 71, 72.

Inactivation of the mouse AFF1/AF4 gene by homologous recombination led to growth defects and revealed that AFF1/AF4 is critical for normal lymphocyte development, but not for other cell types in the haematopoietic system 73. A missense mutation in mouse AFF1/AF4 was recovered in a screen for mutations affecting neuronal cell death and survival 74. Mice carrying this mutation show growth retardation and also have a distinctive jerky ataxic gait, apparent 3–4 weeks after birth due to impaired motor and balance functioning. Histological analysis of brain sections revealed that there is a degeneration of Purkinje cells in the cerebellum from three weeks, leading to significant atrophy of the cerebellum by six months 75.

The *lilliputian* (*lilli*) gene encodes the single AFF-like protein found in *Drosophila*, so is the best fly equivalent to AFF1/AF4. Alleles of *lilli* were first isolated in a screen for factors that suppress activated Raf in eye development (referred to as *Su(Raf)2A*; 76). It has been subsequently recovered from other genetic screens for factors interacting with Ras signalling 77–80, Dpp/BMP signalling 81 and Wnt signalling 82 in addition to screens for maternal mutations affecting segmentation 83, 84. Thus *lilli* plays multiple roles during *Drosophila* development.

*Drosophila* contains a single member of the ELL family of proteins, which is encoded by the *Su(Tpl)* gene 85. Mutations in *Su(Tpl)* lead to embryonic segmentation defects and genetically interact with the Ras signalling and Notch signalling pathways 78, 85–87.

## Insights into proximal pausing control from animal studies

Mutations recovered in genes encoding elongation factors have revealed their involvement in a diverse range of biological pathways including the pathogenesis of numerous cancers, embryonic patterning, haematopoiesis, and also neuronal development, migration and degeneration. However, we are only beginning to appreciate the contribution that promoter proximal pausing and control of P-TEFb activity has during animal development and adult life.

The core components regulating elongation are expressed in all cells and recruited to numerous genes; however, disruption of their function in vivo leads to distinctive defects revealing that promoter proximal pausing must be influenced by contextual transcription factors. A number of proteins have been shown to promote P-TEFb recruitment to stimulate transcription at specific target genes including heat shock factor, Tat and c-Myc 9, 68. However, little is known about which factors interact with DSIF and NELF to regulate pausing. Loss of NELF does not result in a simple global effect on transcription 42, 51 so additional factors must determine which genes are induced or repressed in the absence of NELF.

## Conclusions

Promoter proximal pausing is not an absolute requirement for either rapid or high induction of gene expression, but appears to be a common feature at genes that are normally expressed at some basal level, but which have the capacity to be rapidly induced by changes in cellular environment. Expression of such genes requires very precise control as too little expression may render the cells unable to respond to incoming signals, and too much may trigger expression of downstream effectors in the absence of the appropriate signal.

Rigorous control of cell division is essential in multicellular organisms during development to generate functional tissues and organs and in adult life to prevent tumours. Excessive stimulation of P-TEFb activity often leads to increased cell proliferation; mutations that increase c-Myc activity or fuse the MLL transcription factor to SEC components have been isolated from numerous cancers. Perhaps the tight control of the P-TEFb checkpoint observed in higher animals has evolved as an additional barrier to deregulated cell proliferation.

Animal studies confirm that correct regulation of promoter proximal pausing is critical for development and health in adult life. Analysis of gene expression changes in ES cells and other cell culture systems may hint at the consequences of disrupting the pausing checkpoint in vivo, but to truly understand the biological relevance and role of pausing in vivo, more genetic studies in whole model organisms are necessary.

## Abbreviations

SEC: super elongation complex
TSS: transcriptional start site
